# Ten simple rules for unbiased teaching

**DOI:** 10.1371/journal.pcbi.1010344

**Published:** 2022-10-06

**Authors:** Dean Mobbs, Sarah M. Tashjian

**Affiliations:** 1 Department of Humanities and Social Sciences, Pasadena, California, United States of America; 2 Computation and Neural Systems Program at the California Institute of Technology, Pasadena, California, United States of America; Carnegie Mellon University, UNITED STATES

University teaching, whether by professors, lecturers, or instructors, draws on a number of skills including communicating and simplifying complex ideas, to inspiring students and teaching critical thinking. The social sciences, from psychology to behavioral economics, and anthropology, are often a joy to teach as the fields inherently attempt to understand ourselves by scientifically interrogating all aspects of human nature. What is not often explicitly considered is that the way we communicate science can come with implicit biases and narrow cultural references. This includes the data we present and how we interpret the data, as well as the identity of the scientists themselves. Attempts to increase unbiased teaching are particularly important for members of majority-status groups. This article draws on the recent increase in adoption of learner-centered teaching approaches, which aim to give students more power over learning. In supporting this goal, we offer suggestions for educators to reduce biased teaching practices and create more inclusive and positive learning environments. These are a selection of important biases to be combatted but are not intended to be all-inclusive. The overarching goal is to encourage teachers to continue on the path toward improving diversity and inclusion and to provide concrete action steps to do so.

The aim of this article is to identify the unintended biases that educators can introduce into their classrooms. We discuss a range of seemingly small missteps that have substantial influence on the learning environment and students’ motivation and self-worth. The authors note that these Rules apply to teaching at all stages, from elementary to medical school, as well as across disciplines. These biases may be unrecognized because they are implicit or because they function as an unintended product of cultural understanding. We believe that the examples we give can have a big impact on students’ self-esteem and mold their self-perception. It is the obligation of the teacher to work toward preemptively addressing these biases in the aim of improving the learning experience. We draw from the social sciences to take a step back and look at how and what we teach. Our focus is on social science because it is a subdiscipline that is particularly vulnerable to a lack of representation in terms of the examples used and stereotypes perpetuated. However, all scientific disciplines can suffer from biased teaching.

The hope of this article is that these Ten Simple Rules will guide the teacher away from biases and toward empowering students to help them fulfill their potential. This work was inspired by ongoing discussions between the authors and their students about how to make the classroom a more inclusive environment. We link examples provided by students to social science theories to provide a framework for why violating these Rules can reflect bias and hinder learning. We have organized these Rules into 2 main categories: (a) identifying and addressing bias in teaching practices (Rules 1 to 5); and (b) ensuring a positive and inclusive learning environment (Rules 6 to 10). Our goal is to highlight the students’ perspective and accompany each Rule with concrete actions the teacher can take to address biases undergoing current scrutiny while create positive practices to carry into the future.

## Rule 1: Increase representation of historically minoritized individuals in teaching materials

Presenting data from predominantly white male scientists may create an exclusionary environment that prevents engagement from marginalized students. Tajfel and Turner’s Social Identity Theory [1] posits that our perception of ourselves is determined by our group membership—if no one in the “successful scientist group” is like me, what leads me to believe I belong in that group? This feeling of exclusion includes race, age, disability, class, sex, and nationality.

It is easy to present studies by those who are “most famous.” When presenting examples of early work in the field of social psychology, for example, it is inevitable that white Ivy League male professors will dominate. Citing these studies is fine, but remember that if you solely show these studies, you are making the implicit statement that it is only white males that do this type of research. For example, teaching a class in physics and discussing the work of Einstein, Newton, and Feynman implicitly teaches black and female students that people like them do not, and perhaps cannot, have careers in physics or related fields—or at best that if those students do make inroads in white, male-dominated fields that their work will not be of equal value. A recent study by Storage and colleagues shows that both adults and children implicitly associate brilliance with men more than women [2]. As those authors aptly state, “*More men than women occupy prominent positions in fields that are perceived to require brilliance… When exposed to these gender-imbalanced distributions*, *people may infer that men are simply better suited for careers that require intellectual firepower*.” Indeed, stereotype threat predicts that in such situations, this would confirm the stereotype that females are not good in science, technology, engineering, and mathematics (STEM) domains. For example, Nobel Prize statistics for physics show that the Nobel has been won by 215 males and only 4 females. The push toward diversity and inclusion in STEM has been critical in changing the male–female imbalance in science, yet it is still lagging. The goal of the teacher should be to not only teach the topic in hand, but also inspire students despite the status quo.

### Action steps

Present data from scientists with diverse gender, race, and ethnic representation. Take this opportunity to showcase the great achievements of individuals from historically marginalized groups including that of Katherine Johnson, Donna Strickland, Emmett Chappelle, Mae Jemison, Frances Arnold, Joni Wallis, and Ben Barres. Such examples will act as inspirational role models while also conveying great science. Invite individuals of many different backgrounds to give guest lectures, including those who can speak to experiences like being a first-generation college student. The ability for students to engage with real examples of professionals that look like them and come from varied backgrounds can be a profound experience that furthers pursuits of the field and feelings of inclusion.

## Rule 2: Use representative images and examples, but avoid stereotypes

When giving examples of human behavior, it is inevitable that the teacher will use stock photos to add to the visual impact of the presentation. It is easy to go directly to Google images and pick the first image. As discussed by Safiya Nobel, Google and other search engines have received public criticism for stereotyping and bias in search results [3]. Google has recently rectified some of these issues, although much remains to be done. For example, a search of “people” reveals mainly white, young people and shows no results for people with disabilities or of nonbinary presenting individuals on the first pages. The onus falls on the teacher to be aware of and to add more diversity in class presentations. Providing positive examples of disenfranchised groups is also critical. If you are discussing the topic of violent crime and you only consider black criminals or are discussing parenting and show only female caretakers, you are reinforcing problematic and inaccurate social stereotypes. Creating a curriculum and class environment that is inclusive and representative permits more opportunities for engagement, provides positive interactions, and builds the foundation for educational trust.

Another major criticism that has stretched across fields from sociology, economics, and psychology is that most data and models are based on what is termed “WEIRDs,” or Western, Educated, Industrialized, Rich, and Democratic samples (see Rule 5). Further, most experiments are conducted by male WEIRDs who can introduce biases by imposing their world view and often implicitly creating experiments and models that extend from their egocentric perspective. The teacher must always provide alternative interpretations and critiques of data that could have harmful impact to marginalized groups (see Rule 4). Further, studies using WEIRDs can exclude students from connecting to the implications as “the study does not represent me or how I would act in that situation.”

### Action steps

Present more representative images and studies. A helpful resource for inclusive images is the repository curated by Better Allies, which lists sites that specialize in stock photos and illustrations featuring individuals from underrepresented groups such as non-white individuals, individuals with disabilities, and older individuals [4]. If there is a lack of representative studies for a given topic, engage students in a thought experiment as to how they might better conduct this sort of study or where they think the implications are limited for their identity groups.

## Rule 3: Avoid biased interpretations of research

In 2020, the journal *Nature Communications* published an article that proposed that a principal investigator’s gender plays a role in the success of students. The paper undermined female mentors and was founded on problematic methods and was subsequently retracted. Science should not be prone to political or partisan interpretations and should always aim for unbiased outcomes. Yet in the case of that paper, the results were damaging to female scientists. Consideration of the dimension of potential harm is necessary, particularly when research conclusions have policy implications. For example, neuroscientific studies on sexual orientation have been used in serious policy debates ranging from supporting that sex is dimensional or nonbinary to theories that homosexuality is a mental disorder resulting in egregious persecution such as medical castration. If we talk about such studies, we must steer toward progression, compassion, and correct the discriminatory actions of the past.

### Action steps

Encourage students to consider how appropriate controls or double-blind studies might mitigate biases from the authors of presented studies. Raise questions when presenting socially charged research that expand interpretations beyond what the authors or contemporary critics may have raised. For example, “why is this research important,” “are there other interpretations for the study conclusions,” “what are the policy implications of this work,” and “are the authors’ conclusions justified based on the science.”

## Rule 4: Disentangle sex and gender

Experiments in psychology and neurobiology have historically focused on biological sex assigned at birth by comparing males versus females or representing only one sex. Explicitly and frequently state that people are free to self-identify their gender and express their gender in ways not confined to traditional binary divisions. When presenting experiments that have important sex effects, reiterate that psychology and other sciences have historically focused on males versus females in terms of hormones and neurobiology, so this is why you are presenting sex as a binary construct. Draw sharp distinctions between the research you present and the personal effects that exclusionary constructs and language can have on the students in your classroom. The Parents for Lesbian and Gay (PFLAG), the first organization for lesbian, gay, bisexual, transgender, and queer (LGBTQ+) people, their parents, and families, provides an up-to-date glossary of relevant terms [5].

In addition to considering the roles of sex, gender expression, and gender identity in research, it is important to use appropriate pronouns when teaching (see also Rule 8 on semantics). If a mistake is made with regard to a student’s pronouns, keep apologies quick and polite. Bonnie Ruberg suggests that exaggerated apologies or dwelling on clarifying pronouns can make students feel even more uncomfortable and awkward [6].

### Action steps

If you discuss studies examining sex differences, you should also discuss what the findings might mean for gender and ways in which we can include LGBTQ+ individuals in future research. Consider whether there are parallel studies on gender that might shed light on conventionally described binary sex differences. We suggest it can be helpful to introduce your pronouns at the start of the course and ask students if they want to confidentially share their pronouns. However, we note that some have suggested publicly using non-cisgender pronouns can be uncomfortable in groups of strangers. Thus, an alternative option is to use expression-inclusive pronouns such as they/them/their by default. If you accidentally misgender someone, simply apologize and move forward.

## Rule 5: Adequately communicate and preempt controversial topics

Controversial topics are a critical part of teaching, but one can often walk a tightrope of enlightening the audience and being perceived as biased. Teaching controversial topics allows the teacher to enlighten students and strongly argue for alternative interpretations. The order of these techniques matters. A mistake the teacher can make is to present the topic before the critique. This can cause harm that is harder to repair after the fact. If the audience does not know that you are going to critique the controversial topic, they may feel anxious or stereotyped before you have the chance to address the fundamental problems with the controversial topic. Psychological research such as attentional blink (a phenomenon that suggests there is a limitation in the ability to deploy visual attention) suggests that highly arousing or threatening words can cause people to attentionally miss subsequent stimuli. This argues for giving prominence to discrediting problematic or outdated information—rectifying misconceptions is more important than accidentally representing the controversy as valid.

### Action steps

Preempt the audience by explicitly stating the controversy (i.e., write on the introduction slide rather than verbally presenting) or use trigger warnings. Explicitly dispute problematic or outdated information. Do not assume that students will know you disagree with a stereotype or controversial point if you do not make it abundantly clear.

## Rule 6: Small things can have big effects

The little things matter. Despite good intentions, subtle communications between the teacher and students can have a profound effect. One example of how a seemingly small communication can become problematic comes from work on mindsets. Mindsets are assumptions that shape how you make sense of the world and yourself. Mindsets about one’s abilities can be influenced by subtle language and other small behaviors. For example, it may seem innocuous to praise students as “smart” but associating achievement with intelligence can harm students who are vulnerable to a fixed mindset, as highlighted in work by Carol Dweck [7]. A fixed mindset is an assumption that an attribute, like intelligence, is stable and does not change. Spotlighting intelligence and conveying to students that this quality can be measured from classroom performance can make it harder for students to cope with setbacks. Instead, when teachers praise students for “effort,” setbacks are more easily tackled. Work by Destin and colleagues demonstrates that socioeconomic status (SES) is associated with mindset such that students of higher SES have fewer fixed mindset beliefs [8]. What might seem to the teacher like minor semantics can transcend into something that not only impacts students in both conscious and unconscious ways but can also contribute to existing socioeconomic achievement gaps.

Another prominent example is subtle stereotypes regarding one’s group membership (i.e., race, gender, sexual orientation, and SES), which can directly impact students’ self-perception and ambition. Work by Cohen and colleagues [9] on stereotype threat shows that if a standard test is framed as a test of intelligence, this has a large negative effect on black, compared to white, students because of presumptions about the underlying intelligence of non-white individuals. Subtle changes in framing, representative presentation, and inclusive language can eschew assumptions about stereotypes or diversity to foster a respectful classroom environment. A respectful classroom establishes the base from which to challenge students to use their voice and experiences to advance the collective discourse. Discrimination is layered and although there has been societal progress, the residual effects are still here and there is still immense room for improvement.

While this Rule overlaps with Rule 8, the critical difference is that in Rule 6, the influence of interactions is determined by the receiver, not the transgressor. In 2015, Simba Runyowa wrote an article for The Atlantic about microaggressions and why they matter [10]. Runyowa defined microaggressions as “behaviors or statements that do not necessarily reflect malicious intent, but which nevertheless can inflict insult or injury.” It is the impact of the statement that is the key aggressor, despite the intent of the actor. Following Rule 8 regarding changing semantics can help make headway toward achieving the intention of Rule 6 and minimizing negative effects of seemingly small transgressions. However, semantic updates alone are not sufficient. The teacher needs to start from a place of empathy, recognizing that the balance of power in the classroom has consequences on the weight of their actions.

### Action steps

McKenna Princing provides some tips on preventing microaggressions that can also mitigate stereotype threat and fixed mindset assumptions [11]. A common theme to these tips is to first look within yourself to understand what inputs and preconceived beliefs motivate your initial response to differences. After reflecting on how you perceive the world, transition to understanding how your actions are perceived and make a concerted effort to respond to others with empathy.

## Rule 7: Avoid inappropriate jokes and images

All teachers want to make their class fun. Humor is often a key part of the great speaker’s repertoire. Humor can make a mind-numbing topic enjoyable and re-memorable. Yet, jokes can vary from the innocent to the mean, and there is large variation across students in what is considered appropriate. Jokes can take the form of a simple GIF, cartoon, or be spoken, and each can be offensive. In these politically heated times, jokes about political figures are common, yet may offend some people in your audience. If your intent is to use humor to engage students, you have to be careful about jokes that only invite some students to laugh along. If students feel like they are not fully welcome or do not fully belong in their own classroom, you are creating two different learning environments. Students should not have to endure a personal cost to be where they have the right to be. Although light humor might be justified in certain contexts, it is important to preserve the safety of the classroom for students.

Beyond the types of triggering jokes and images that may be readily apparent, there are important sensitivities with respect to intersectionality that must be considered. Gracefully embracing a diverse audience may be a difficult or daunting task. Continual self-reflection and openness to feedback can help improve teaching.

### Action steps

Stay away from political, sexual, sexist, racial jokes, jokes about nationality or disability. Ask yourself the simple question, “is this joke important to communication of the topic?” If not, “could the joke offend someone in the audience?” If the answer to the second question is ambiguous, remember that even innocuous intentions can create a hostile atmosphere for some students. Seek out anonymous feedback from colleagues and students to gauge the success with which sensitivity to intersectionality is achieved in your classroom.

## Rule 8: Familiarize yourself with changing semantics

Semantics is the study of meaning and typically focuses on the association between signifiers, such as words, and what they represent. When those words represent people, the importance in updating semantics in teaching is unquestionable. In the 1990s, the *American Journal of Mental Retardation* changed its name to the *American Journal of Intellectual and Developmental Disabilities*, due to the now obvious negative connotations (see Rosa’s law; [12]). Although the journal had existed since 1944, it was time to retire the old title and move forward with more progressive semantics. In recent years, parallel changes have occurred with descriptions of gender and race. As discussed in Rule 4, terms such as nonbinary, pansexual, and transgender should be used in the appropriate context [13]. Likewise, calling a black individual an African American is increasingly under debate. Similarly, terms like non-white are seen as too broad and exclusory. Updated semantics acknowledge that black individuals have meaningfully diverse and nuanced ethnic and racial identities [14], while acknowledging that previously accepted terms are offensive [15]. Teachers should familiarize themselves with appropriate semantics, but also recognize that debate is ongoing with regard to many gender and racial identifiers.

Adopting changes in semantics is critical for ensuring inclusivity and respecting students from diverse backgrounds and intersectionalities. When student disagreement or criticism arises, teachers should respectfully listen, adopt currently accepted semantics, and acknowledge the limitations of outdated semantics in assigned readings or other works. The ultimate goal is to reduce stigma and burden on students in the learning environment (see Rules 7 and 9).

### Action steps

Teachers are not alone in navigating changing semantics. Products are now available to encourage use of inclusive language including a file of phrases and responses [16] provided by Rands Leadership Slack group. This list of phrases can be used as part of Slack to generate automatic explanations of why the word or phrase is not inclusive as well as alternatives to consider. Sensitivity training with respect to the use of inclusive language can help teachers feel supported in their desire to cope with an evolving semantic landscape. The key is to seek out information and remain open to suggestions from students and colleagues as to how changing semantics affect others (see also Rule 10).

## Rule 9: Provide positive, kind, inclusive, and encouraging feedback

The bidirectional nature of teaching is reflected in teachers posing questions that students answer, students proposing ideas that teachers critique. However, what is perhaps less obvious during these exchanges is the opportunity to exhibit empathy and compassion in a public forum. The teacher should be consistently aware of the how they provide feedback to students. When students raise their hand to answer a question, state how many responses you will take and mindfully call on students who less frequently engage in class discussion, considering representations of gender and race. Considerations of intersectionality are of increased importance, with data showing bias in teachers’ perceptions of even young children’s abilities as a function of race and gender [17]. Giving students visibility can ameliorate feelings of stigmatization and being ignored while increasing individual agency and intersectionality in the classroom.

When students are in supportive learning environments and their sense of self-efficacy is high, motivation is amplified [18]. Increasing motivation can support the retention of underrepresented students in STEM and higher education. Thus, the implications of positive and supportive feedback are profound. Research by Stanford psychologists Geoffrey Cohen and colleagues investigated how buffering critical feedback given to students helped close racially divided performance outcomes. When feedback was accompanied by an assurance of the student’s capacity to reach the teacher’s high standards (e.g., statements like “I have high standards and I know you can meet them”), black students responded as positively as white students, engendering trust rather than stereotype threat. When later deployed in a sample of middle school students, effects were particularly strong for black students who felt mistrusting of schools, with only 17% of a nonintervention control group choosing to revise a submitted paper after feedback, but a huge 71% of black students who received the reassuring feedback choosing to revise their papers. Thus, positive feedback can alter student motivation, self-worth, and, consequently, academic outcomes [19]. Educators play an important role not only in disseminating information, but also in shaping students’ feelings of self-worth and trust in educational institutions by providing self-affirming feedback and reducing bias in class.

Research on implicit bias suggests humans tend to implicitly confirm stereotyping thoughts or beliefs. This may manifest in varied ways from body language to the way we talk about or to students. Influential work by Stanford Professor Jennifer Eberhardt has shown how language of police officers is more negative when interacting with black compared to white community members [20], and how school discipline is harsher for black students [21]. Professor Eberhardt’s work also has implications for other groups and suggest that we need to be aware of these and other biases that can have a large impact on students’ self-identity and confidence.

### Action steps

Become that teacher who inspired you. Constructive feedback, positive tone, openness, and encouragement can have a meaningful impact on students and their educational experience. The goal is ultimately to motivate and help students. Eschew assumptions about how difficult or easy assignments or topics may be. Use motivating language and effort-based praise to encourage students to identify their personal goals for the course and actively work toward them.

## Rule 10: Be open and embrace change

At a meeting in Boston, Harvard Professor Ellen Langer uttered: “*I like to be wrong because I learn something new*” [22]. A critical part of an open and progressive society is to embrace the reality of continuous learning and evolution. Calls for improving biases can sometimes feel like criticism and prompt defensiveness. Defensive responses often trigger the same response in others. Instead, seek out ways to be more solution-oriented by listening to advice from others and building upon it. You will soon find yourself on your way to more cooperation and collaboration. Be open to guidance from others. It is okay to make mistakes, to say the wrong thing. But it is more important to identify and correct those mistakes.

What was accepted 20 years ago is, in many cases, no longer acceptable, and it is our responsibility as educators to continue educating ourselves on the changing cultural landscape. We have all heard older generations use derogatory terms, and like Ron Perlman’s character, Benedict Drask, in the movie “Don’t Look Up,” we do not want others to say we are “*from a different generation*” [23]. The preventative tonic here is to recognize when and where your expertise is limited. You may be the neuroscience expert in the room, but that does not preclude the opportunity to listen to and learn from your students and the broader community when it comes to improving the inclusiveness of your teaching practices.

Behavioral economics provides us with examples of biases that pushes against this Rule. The “conservative bias” dictates that one will not revise their beliefs even when presented with contrary evidence [24]. Self-awareness and accountability are crucial for combatting this bias to more accurately integrate new information into our existing knowledge schemas [25]. Another is the “status quo bias” where people prefer not to change [26]. Inertia can prevent people from attaining desired outcomes, including creating considerate and inclusive classrooms. Despite widespread acknowledgment of bias, social scientists often fall foul of these and other cognitive errors that hinder attempts to be open minded. Explicitly acknowledging that these biases are ever-present and can have harmful effects is a first step to actively embracing change.

### Action steps

Continue learning about how best to overcome one’s inherent biases by attending sensitivity training or other workshops on cognitive biases. Private organizations like Better Allies and Turbine Labs [27] provide actionable challenges and ways to address biases in the workplace as well as briefings on challenges in the DEI (Diversity, Equality and Inclusion) landscape.

### Additional biases that can invade teaching

This set of Rules is not intended to be exhaustive but is a starting point for educators to examine how their actions can shift the climate of the classroom. In the spirit of inclusivity, we compiled a list of 10 additional important examples of biases that were not discussed in detail above.

### 1. Expertise

As teachers gain more expertise in their field, they may forget how dense or intractable information was when first learning. This “curse of expertise” is a cognitive bias where the individual who is communicating assumes others have the background knowledge to understand. It is important for the teacher to monitor their own expertise bias as well as the communications from students or guest lecturers regarding material that may need clarification to bring everyone to the same foundational understanding [28].

### 2. Visual access

One in 12 males and 1 in 200 females are color blind. Using color blind–accessible color palettes when making figures and slides is a simple but important way to make learning materials accessible for individuals with visual impairments.

### 3. Physical limitations

Be cautious when proposing activities that require mobility. As one Reviewer noted, they once proposed an activity where students needed to use both hands but one student was missing an arm. Take efforts to increase inclusion by being mindful of physical mobility limitations early on. One way to assess physical limitations is to provide students an opportunity to directly share accessibility issues confidentially so you can tailor later exercises appropriately.

### 4. Financial burdens

Learning about the cost of materials required for classes and making materials more accessible through electronic options or direct dissemination can alleviate stressors for students with low financial means.

### 5. Learning disabilities

Learning disabilities are not only relevant at the time of exams/assignments. In each class, breaking learning tasks into small steps and regularly checking understanding can help students with disabilities more effectively learn. Present information visually and verbally, use diagrams and graphics to support spoken instruction (see also #6 below).

### 6. Language

Avoid equating lower proficiency with spoken English with poor writing skills or diminished learning ability. Be aware that non-native English speakers may find it hard to understand a native English teacher and feel excluded if they lose some of the teaching material. Providing recordings with closed captioning can help non-native English speakers return to material later.

### 7. Tokenization

An assumption that international students have expertise or interest related to their race, ethnicity, or home country runs the risk of tokenizing these individuals. Similarly, do not presume individuals from underrepresented groups are comfortable speaking on behalf of that group or sharing personal experiences with discrimination.

### 8. Historical context

Initiating discussions on sensitive topics without realizing certain students may not have the relevant historical and cultural context can cause some students to feel excluded. For example, racism in the United States has a unique history compared with racism in Asia, which informs much of the current political and social discourse about stereotypes and discrimination. Make sure all students have any necessary context before engaging in important discussions.

### 9. STEM stereotypes

Providing more or less support to students based on beliefs that some groups are not “innately talented” may be difficult to detect because it may not be based on overt stereotypes. Provide support to the class as a whole and offer multiple avenues through which students can access that support (teaching assistants, supplemental materials available online, slack channels to connect students in study groups, etc.). See additional reading by Niral Shah on how even seemingly complementary STEM stereotypes are problematic [29].

### 10. Dismissive/Demotivating statements

Use of unintentionally dismissive language by suggesting something is “easy” or that students should “already know this” can be demotivating. Similarly, prompting students to think the course will be insurmountably difficult demotivates learning (10, Chapter 3). Be mindful not to suggest that an assignment or topic is simpler or easier to accomplish as this might prime students to think that they are “behind” or “incompetent.” On the flip side, suggesting a course is extremely arduous can compromise expectations for success and undermine motivation. Create a classroom that welcomes mistakes, questions, and opportunities for growth rather than amplifying insecurities and external comparison.

## Conclusions

As Ambrose and colleagues astutely state, “*Students’ current level of development interacts with the social*, *emotional*, *and intellectual climate of the course to impact learning*” [16]. Our hope is that these Rules will prompt further discussions with students and colleagues to create more empathetic and constructive learning environments.

Writing these 10 Rules has been enlightening and gave the authors time to think deeply about how we present data, what data we present, and by whom. We need to continue progressing with society, respecting new views and changes, and actively adapting when we fall short. We all have the duty not only to stay inclusive and tolerant, but to also be aware of our own biases. Challenge yourself to let these 10 Rules guide you to critically examine your teaching and language practices. In this pursuit, ask yourself if you are addressing the biases you bring to your classroom. Set goals for yourself on how to raise personal awareness and elevate your students. To finish with a quote from Dr. Martin Luther King Jr, “*If you can’t fly then run*, *if you can’t run then walk*, *if you can’t walk then crawl*, *but whatever you do you have to keep moving forward*.”
